# Prevalence of Multidrug-Resistant Bacteria (Enteropathogens) Recovered from a Blend of Pig Manure and Pinewood Saw Dust during Anaerobic Co-Digestion in a Steel Biodigester

**DOI:** 10.3390/ijerph20020984

**Published:** 2023-01-05

**Authors:** Christy Echakachi Manyi-Loh, Anthony Ifeanyin Okoh, Ryk Lues

**Affiliations:** 1 Centre of Applied Food Sustainability and Biotechnology (CAFSaB), Central University of Technology, Bloemfontein 9301, South Africa; 2 SAMRC Microbial Water Quality Monitoring Centre, University of Fort Hare, Alice 5700, South Africa; 3 Department of Environmental Health Sciences, College of Health Sciences, University of Sharjah, Sharjah P.O. Box 26666, United Arab Emirates

**Keywords:** gram negative bacteria, environmental sources, antibiotic resistance, multidrug resistance phenotypes, South Africa

## Abstract

South Africa adopts intensive livestock farming, embracing the employment of huge quantities of antibiotics to meet the increased demand for meat. Therefore, bacteria occurring in the animal products and manure might develop antibiotic resistance, a scenario which threatens public health. The study investigated the occurrence of Gram-negative bacteria from eighteen pooled samples withdrawn from a single-stage steel biodigester co-digesting pig manure (75%) and pine wood saw dust (25%). The viable counts for each bacterium were determined using the spread plate technique. The bacterial isolates were characterised based on cultural, morphological and biochemical characteristics, using the Analytical Profile Index 20 e test kit. In addition, isolates were characterised based on susceptibility to 14 conventional antibiotics via the disc diffusion method. The MAR index was calculated for each bacterial isolate. The bacterial counts ranged from 10^4^ to 10^6^ cfu/mL, indicating manure as a potential source of contamination. Overall, 159 bacterial isolates were recovered, which displayed diverse susceptibility patterns with marked sensitivity to amoxicillin (100% *E*. *coli*), streptomycin (96.15% for *Yersinia* spp.; 93.33% for *Salmonella* spp.) and 75% *Campylobacter* spp. to nitrofurantoin. Varying resistance rates were equally observed, but a common resistance was demonstrated to erythromycin (100% of *Salmonella* and *Yersinia* spp.), 90.63% of *E*. *coli* and 78.57% of *Campylobacter spp*. A total of 91.19% of the bacterial isolates had a MAR index > 0.2, represented by 94 MAR phenotypes. The findings revealed multidrug resistance in bacteria from the piggery source, suggesting they can contribute immensely to the spread of multidrug resistance; thus, it serves as a pointer to the need for the enforcement of regulatory antibiotic use in piggery farms. Therefore, to curb the level of multidrug resistance, the piggery farm should implement control measures in the study area.

## 1. Introduction

South Africa has profound interest in livestock farming since it contributes immensely to its socio-economic capacity, creating jobs for the population, most especially to the individuals in the poverty-stricken rural communities [1]. Consequently, 70% of its agricultural land is employed in livestock farming with the distribution in numbers, breeds and species throughout all the provinces being influenced by grazing, environment and production systems [2]. Livestock farming generates copious quantities of manure containing significant organics, nutrients, heavy metals, antibiotics and pathogens; however, the heavy metals and pathogens can result in serious environmental pollution [3].

Specifically, in South Africa, the province of Eastern Cape is viewed as the poorest province amongst all the provinces of the country following its demographic, health and socioeconomic patterns [4]. Owing to its high level of food insecurity, the local people depend on natural resources for daily living and subsistence. According to Ngumbela et al. [5], the food insecurity challenge can be addressed via agricultural productivity. Therefore, some local inhabitants may utilise raw manure as fertiliser on farms to promote the growth of plants and crops; this is because animal manure is regarded as the oldest and a universal fertiliser and as a source of humus, micro and macro nutrients, useful organisms, which indirectly and positively affect the chemical, physical and biological components of the soil when applied on agricultural lands [6]. Moreover, Blaiotta et al. [7] noted that livestock manure constitutes a variety of pathogens, of which some are pathogenic causing infections in humans, whereas others are highly host-adapted and not pathogenic to humans. The accidental or deliberate release of the manure (containing microbes and other chemical contaminants such as antibiotics) into the environment and water bodies might occur through uncontrolled application of animal manure onto land, heavy rainfall wash offs and by leachate seeping through the soil from heaped/stockpiled manure on farms where composting is allowed [6]. 

Globally, antibiotics are employed in animal farming for several reasons, including growth promotion, treatment and/or prophylaxis. However, there appears to be a variation in the association between antibiotic use in the livestock industry and meat production in European countries, the United States and in other nations of the world [8]. Precisely, in developing countries, Van et al. [9] pointed out that the use of antibiotics in the food industry is to promote the wellbeing and growth of the animals and such a practice provides a host of economic benefits to the producers and the consumers in general. Similarly, Meissner et al. [10] highlighted that in South Africa, livestock farming is practised either as intensive farming to ensure sustainability or associated with communal and rural farming, but, on the whole, livestock is kept owing to its contribution to food security, sustenance and the economy. Despite these positive contributions, livestock farming is associated with serious negative environmental and health impacts, including climate change effect (global warming), damage to the ecosystems, reduction in biodiversity as well as pollution that might result in antibiotic resistance through animal manure, animals and animal products [11]. 

It is worrisome as some of the veterinary antibiotics utilised are similar or surrogates for those employed in clinical settings for the treatment of humans [10]. The frequency of use of antimicrobials in food production directly mirrors the level/degree of emergence of resistant foodborne pathogens. However, the overuse or the frequent use of the antibiotics administered either to the animals for treatment/prophylaxis and/or included in small quantities in their feeds leads to constant exposure to these molecules, thus creating selective pressure that can ultimately lead to the selection and/or emergence of antibiotic-resistant microorganisms [8]. In addition, antibiotic resistance of microorganisms found in animals, animal wastes and animal products cannot be secluded as these organisms can eventually be transmitted through the food chain, direct contact with animals and the environment to humans by contamination/pollution [12]. Consequently, antibiotic resistance has been regarded as one of the major serious public health threats confronting humanity [11]. 

More elaborately, there are plausible routes through which antibiotic-resistant bacteria can reach humans or the environment; administered drugs are being excreted and can remain unaltered in the environment [13], and, during slaughtering, gastric lavage might occur, a situation wherein the intestinal contents are spilled over the meat products. Slaughterhouse processing of infected animals as well as animal husbandry staff and farm workers with a greater probability of developing resistant infections may in turn disseminate these resistant bacteria through meat and even the staff themselves to the population at large [10,12]. It is of no doubt that the environment harbours bacteria that are resistant to antibiotics and, naturally, bacteria can develop resistance to antibiotics over time [10]. Nevertheless, the food industry is fraught with a fundamental challenge, which seems to be antibiotic-resistant bacteria occurring in the food chain [8]. The presence of antibiotic resistance in humans can cause difficult to treat infections, long hospital stays, high treatment costs, less sustainable production of food causing food shortages, serious treatment side effects owing to the employment of the last line antibiotics involving the increasing use of broad-spectrum antibiotics and, finally, increased morbidity and mortality [14,15]. 

It is worth mentioning that testing the susceptibility of bacteria to antibiotics helps in the management of infections by clinics, hospitals and national programmes for the control and prevention of infectious diseases. In particular, antibiotic susceptibility testing has great impact on a patient’s management via identifying the specific diagnosis and targeting the specific disease-causing agent responsible for the disease condition [16]. Notwithstanding, antibiotic susceptibility testing has been implemented continuously in surveillance activities for resistance patterns in bacteria. Data have demonstrated that the antibiotic susceptibility profiles of bacteria vary with time, the individual and the geographical area owing to mutations in the bacterial DNA and the possibility of transmission of resistance genes through horizontal gene transfer from one bacterium to another [17].

Interestingly, Leopold et al. [18] noted the high disease burden in Sub-Saharan Africa that includes bacterial infections (diarrhoea, pneumonia, typhoid, sepsis, sexually transmitted diseases) caused by the bacteria of the Gram-negative category, and they are said to be the leading cause amongst others of infections/diseases and deaths occurring throughout the region. Furthermore, in this region, antimicrobial resistance (AMR) is exacerbated following the imprudent use of antibiotics that are bought over the counter without appropriate prescription, scarcity of clinical microbiology laboratories that perform sensitivity assays, the non-existence of a harmonised AMR observatory and frail regulatory frameworks for the exposure and implementation of antimicrobial agents added to the endorsement of the use of antibiotics by a great portion of the population having the human immunodeficiency virus to eradicate opportunistic diseases, a practice that has aggravated the emergence of resistant pathogens [18,19]. Against this background, it is apparent to investigate possible sources of origin and the distribution of antibiotic resistant bacteria in a bid to generate the information necessary to update the existing national and international database of antibiotic resistance. Such findings will be relevant in developing disease control measures, in policy making as well as in improving the principles guiding effective antimicrobial stewardship [20]. In this light, the study was conducted to screen manure samples and the co-digesting mixture (pig manure and pine wood sawdust) during anaerobic digestion for the occurrence of bacterial pathogens (Gram negative) of known environmental and public health concern, to elucidate their antibiotic resistance patterns as well as to determine the multiple antibiotic resistance indices and phenotypes. 

## 2. Materials and Methods

### 2.1. Sampling

Each sampling entailed the collection of multiple samples (between 5 and 7 mL) from different sites of the biodigester following stirring to ensure consistency, homogeneity and even distribution of the microorganisms throughout the digesting mixture. Then, the samples were pooled together, representing the sample for each day. Overall, 18 pooled samples (between 15 and 21 mL) were withdrawn from a single-stage steel biodigester that was charged with substrates, comprising pig manure (75%) and pine wood saw dust (25%) in the ratio 3:1 for anaerobic co-digestion. The pig manure was procured from a piggery farm and the saw dust from a sawmill, both located in proximity to the Fort Hare University, Alice Campus, and were used to charge the biodigesting chamber. The biodigesting chamber of 100 L capacity but of 75 L working volume was batch-operated under a psychrophilic temperature range of 13.16 to 26 °C and the samples were withdrawn after stirring for 2–3 min (to mix the contents) with the help of a stirrer inserted into the digester during the designing and deployment of the biodigester. Mixing was done daily, since mixing has prominent effects on microbial community, methane content and volatile fatty acids and mixing at intervals is more preferable [21]. However, samples were only collected every 7 or 14 days over seven months for the evaluation of bacterial counts and the cultivation of enteropathogens of public health concern as per the procedures of Poudel et al. [22]. The overall number of samples constituted both the untreated and the treated biomasses. The untreated biomasses were the original samples procured from the sawmill and the piggery farm while the treated biomass was the mixture, or a blend of the samples withdrawn from the digester during the anaerobic digestion process.

Each sample was collected and introduced into a sterile centrifuge tube containing tryptic soy broth (Liofichelm, Diagnostics, Roseto degli Abruzzi, Italy) and the tubes were placed on ice [23] upon transportation to the laboratory for subsequent processing, bioassays and analysis.

### 2.2. Determination of the Counts of Viable Bacteria

The counts of viable bacteria (four) classified as Gram-negative, including *Escherichia coli*, and species of *Salmonella*, *Yersinia* and *Campylobacter* that are mostly associated with gastrointestinal diseases, were determined using the spread plate technique, performed according to the method previously described [22]. Briefly, each sample was carefully vortexed and 1 mL was transferred into 9 mL of sterile physiological saline (0.9%) in a test tube to prepare an initial 1:10 dilution, which was 10-fold serially diluted to constitute dilutions, including 10^−1^ to 10^−5^. Depending on the bacteria of interest, different microbiological media were prepared following manufacturer’s instructions, which consisted of *E*. *coli* chromogenic agar (Conda, Madrid, Spain), *Salmonella*/*Shigella* agar (Conda, Madrid, Spain), *Yersinia* selective agar base (Conda, Madrid, Spain) and, lastly, modified charcoal cefoperazone desoxycholate agar (Conda, Madrid, Spain). For the growth of *Yersinia* and *Campylobacter* species, the microbiological media were enriched with CIN (cefsulodin, 7.5 mg: irgasan, 2.0 mg: novobiocin, 1.2 mg) supplement and CCDA (cefoperazone, 0.016: amphotericin B, 0.005) supplement, respectively, depending on the volume of media prepared. All the supplements were purchased from Oxoid, United Kingdom. 

While observing aseptic conditions, the solidified agar plates were inoculated by dispensing 100 μL of each dilution and the inoculum spread using a sterile glass spreader. Subsequently, the inoculated plates were incubated aerobically at 37 °C for between 18 and 24 h for the cultivation of *E*. *coli* and *Salmonella* spp., at 30 °C for 24–48 h to enable the growth of *Yersinia* spp. and at 42 °C under a controlled oxygen atmosphere constituting 5% O_2_, 10% CO_2_ and 85% N_2_ (BR0038, Oxoid, UK) for 24–48 h for the growth of *Campylobacter* spp. Presumptively, the bacteria were identified following incubation as follows: dark blue or violet colonies observed on *E*. *coli* chromogenic agar were considered as *E*. *coli* [24], pink colonies with a black centre on SSA were counted as *Salmonella* spp. [25], colourless colonies with a red centre or which appeared with red bull’s eyes were regarded as *Yersinia* spp. [26] and the presence of colourless, tiny, smooth, convex and translucent to grey coloured colonies were enumerated as *Campylobacter* spp. [27]. The bacterial counts were enumerated as colony forming units and the values were recorded as a mean of triplicate assays on respective tables.

### 2.3. Isolation, Biochemical Characterisation and Storage of the Bacteria

After enumerating, well isolated and suspected/presumed colonies on the different agar plates were selected and steaked several times either on freshly prepared sterile nutrient agar (Merck, Modderfontein, South Africa) or Mueller Hinton agar (Conda, Madrid, Spain) plates, to generate pure culture as per the method of Manyi-Loh et al. [28]. 

Oftentimes, subcultures are performed on nutrient agar, a multipurpose and cheap agar. However, when fastidious organisms that need special nutritional requirements for growth are involved, the subculture medium must be chosen carefully. Therefore, nutrient agar was used for the subculture of *E*. *coli* and *Salmonella* isolates while Mueller Hinton agar, a standard medium recommended for antibiotic susceptibility testing, was employed and supplemented with the antibiotics for the growth of *Yersinia* and *Campylobacter* species [29]. This is because Mueller Hinton is a loose agar and permitted a better diffusion of the supplements (antibiotics) throughout the medium, resulting in adequate bacterial growth. 

Purified cultures of each bacterium were harvested and introduced into tryptic soy broth supplemented with 20% glycerol (a cryopreservation agent that inhibits intracellular ice formation during freezing) and were stored at −80 °C as stock cultures [30] for further analysis. Bacterial identification was established by growth on selective microbiological media, morphological characteristics along with biochemical characterisation [31]. The analytical profile index (API) 20e kit was used for the biochemical characterisation of *Enterobacteriaceace,* while other biochemical tests, including the presence of enzymes (catalase, oxidase, urease), fermentation of sugars, susceptibility to nalidixic acid, microaerobic growth at 37 °C and 42 °C and the production of hydrogen sulphide gas (Triple Sugar Iron) and indole test, were employed for the confirmation of the other bacteria. Confirmed isolates were preserved at −80 °C in tryptic soy broth plus 20% glycerol.

### 2.4. Determination of Antibiotic Resistance Phenotypes

The growth inhibition caused by antibiotics was assayed using the Kirby–Bauer disc diffusion technique while employing a collection of 14 traditional antibiotics (Mast, Diagnostics, Bootle, UK). Stock cultures were resuscitated on either nutrient agar (Merck, Modderfontein, South Africa) or Mueller Hinton agar (Conda, Madrid, Spain) considering the species of bacteria. The growth of the bacterial isolates was harvested and employed in the susceptibility testing, which was performed guided by the procedures of the Clinical Laboratory Standard Institute (CLSI) [32]. A standardised inoculum, containing 10^8^ cfu (matching 0.5 MacFarland Standard), was prepared for each bacterial isolate by emulsifying 2–3 distinct colonies from each growth in sterile physiological saline (0.9%) contained in a test tube. Mueller Hinton (Conda, Madrid Spain) agar plates were prepared following manufacturer’s instructions and were allowed to solidify. 

Each solidified plate was swabbed with inoculum-impregnated cotton sticks to generate an even growth pattern of the organism on the plate. The plates impregnated with different bacterial inocula were left for a while to reduce wetness on the plates and then the antibiotic discs were aseptically placed on each plate at equal distance from each other, but not too close to the borders of the plates. This action aided in preventing the zones of inhibition from overlapping following antibiotic action. The discs employed included ampicillin (AMP; 25 μg), gentamicin (GM; 10 μg), chloramphenicol (C; 30 μg), ciprofloxacin (CIP; 5 μg), amoxicillin (10 μg), nalidixic (NA; 30 μg), tetracycline (TET; 25 μg), amoxicillin-clavulanic acid (Augmentin, AUG; 30 μg), trimethoprim-sulfamethoxazole (co-trimoxazole, TS; 25 μg), erythromycin (E; 15 μg), streptomycin (S; 10 μg), nitrofurantoin (Ni; 300 μg), sulfamethoxazole (SMX; 300 μg) and cefotaxime (CTX; 30 μg). The tested plates were incubated at different temperatures and atmospheric conditions based on the bacterium. The examination of incubated plates was conducted to locate zones of inhibition and the diameter of the emergent zone of inhibition around each disc was measured in millimetres and recorded in the respective tables. Each measurement represented the mean of triplicate assays. The interpretation criteria based on the diameter of the zone of inhibition were adopted from CLSI [32], describing the isolate as susceptible or intermediate or resistant. As a positive control, *Escherichi coli* ATCC 25922 was evaluated alongside the test bacterial isolates.

### 2.5. Calculating Multiple Antibiotic Resistance (MAR) Index and Presentation of Their Resistance Patterns

The MAR index can be obtained from the formula MAR= a/b, where ‘a’ denotes the amount of antibiotics that expressed no significant activity against the tested bacterium, being referred to as resistance, and ‘b’ gives the complete antibiotics to which the isolate was exposed in the susceptibility study [33]. An estimated MAR value above 0.2 was indicative of resistance expressed to multiple antibiotics and the bacterial isolate originated from a hypothetically unsafe source, where antibiotics are repeatedly being used, thus a great risk of contamination [34]. Multiple antibiotic resistance phenotypes were developed for all 159 bacterial isolates that were tested against 14 commercial antibiotics and MAR was considered as resistance to ≥3 antibiotics.

## 3. Results

Bacterial counts (colony forming units per millilitres) indicated differences between bacterial species at the time of charging of the digester. Data showed that the co-digesting mixture contained 7.1 × 10^4^ cfu/mL of *Yersinia* spp., 9.0 × 10^4^ cfu/mL *Campylobacter* spp., 2.0 × 10^6^ cfu/mL of *E*. *coli* and 7.0 × 10^4^ cfu/mL of *Salmonella* spp. As the anaerobic co-digestion progressed with time, the bacterial counts of the different species were reduced by 1 log, and *E*. *coli* had the shortest survival period of 77 days, followed by *Salmonella* spp. (84 days) and *Yersinia* spp. (98 days), while *Campylobacter* survived for the longest period of 112 days. Moreover, 18 pooled samples were withdrawn from the digester at 7- or 14-day intervals over a period of seven months. The prevalence rates ranged from 22.22% to 50%, with nine (9) samples found positive for *Campylobacter* spp., five samples (5/18; 27.78%) were positive for *Salmonella* sp. and *Yersinia* sp. alongside four samples (4/18; 22.22%) being positive for *E*. *coli*. In total, 159 bacterial isolates belonging to the genera *Yersinia* (26), *Salmonella* (45) and *Campylobacter* (56), as well as *Escherichia coli* (32) bacterial strains, were recovered from the co-digesting medium (pig manure plus pine wood sawdust) before and during anaerobic digestion taking place in a single-stage steel biodigester. These bacterial species were confirmed into the various genera as follows: *E*. *coli* isolates showed a positive reaction to the presence of the enzymes (lysine decarboxylase, ornithine decarboxylase), fermentation of various sugars (glucose, sorbitol, mannose, rhamnose, melibiose, arabinose), hydrolysis of o-nitrophenyl-b-D-galactopyranoside and the production of indole from tryptophan. Additionally, *Salmonella* spp. fermented various sugars (glucose, mannose, sorbitol, rhamnose and arabinose), producing hydrogen sulphide gas, utilised citrate as the sole source of carbon and hydrolysed o-nitrophenyl-b-D-galactopyranoside. Similarly, *Yersinia* spp. showed the presence of catalase enzymes, fermenting glucose and sucrose with no hydrogen sulphide gas produced, but in the reaction in the indole test, urease and oxidase were both positive and negative, implying we had both negative and positive isolates. Furthermore, *Campylobacter* spp. showed the presence of oxidase and catalase enzymes and exhibited susceptibility and resistance to nalidixic acid. 

Table 1 shows the percentages of sensitive, intermediate and resistant bacterial isolates realised from the sensitivity assay, evaluating the activity of a panel of 14 antibiotics against 159 bacterial isolates. Overall, the susceptibility profiles depended on the tested antibiotics and the bacterial isolates; the maximum sensitivity of the different bacterial isolates included a 100% sensitivity of *E*. *coli* to amoxicillin, 96.15% and 93.33% sensitivity demonstrated by both *Yersinia* and *Salmonella* spp. to streptomycin, respectively, as well as nitrofurantoin’s exhibited effect against 75% of *Campylobacter* sp. In addition, appreciable intermediate activity was exerted by nalidixic acid against 86.67%, 76.92% and 68.75% of *Salmonella* spp., *Yersinia* spp. and *E*. *coli*, respectively. 

On the other hand, the greatest resistance amongst the bacterial isolates occurred against erythromycin, as a common antibiotic, as follows: 100% of *Yersinia* spp. and *Salmonella* spp. and 90.63% of *E*. *coli*, but 98.21% *Campylobacter* spp. displayed resistance to cefotaxime. However, in particular, *Salmonella* isolates showed complete resistance (100%) to two (2) other antibiotics, including tetracycline and amoxicillin. Similarly, *E*. *coli* isolates showed a notable resistance (81.25%) to tetracycline. In addition, *Yersinia* isolates exhibited a profound resistance of 92.31% to sulfamethoxazole. Lastly, *Campylobacter* isolates also presented with a huge resistance to erythromycin (78.57%).

**Figure 1:**
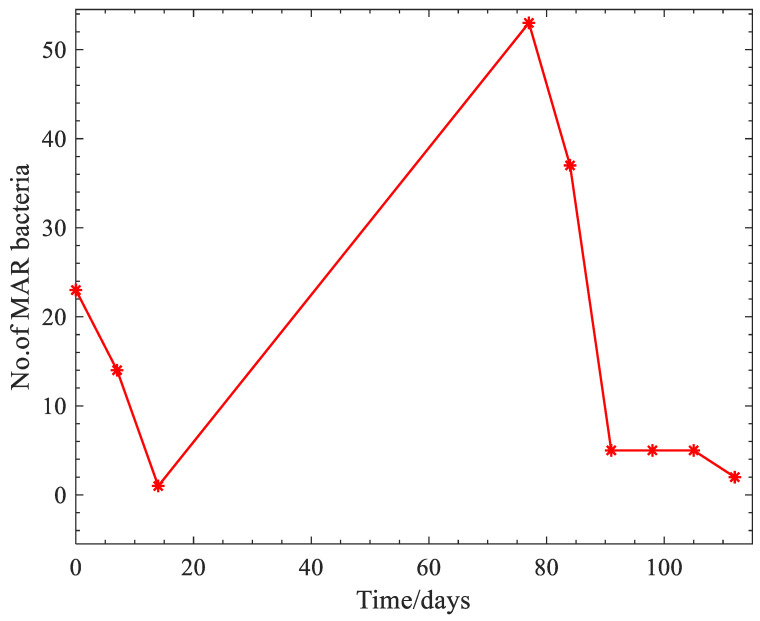
Variation of the number of multiple antibiotic resistant bacteria with time.

From the calculated multiple antibiotic resistance index, it was revealed that 145 bacterial isolates (91.19%) had a MAR index > 0.2, indicating resistance to ≥3 antibiotics and the range of the MAR index for each bacterium is as shown in Table 2. More elaborately, only five isolates (3.45%) had a MAR index less than 0.2; however, two (2) isolates displayed a MAR index of 0.9 (i.e., resistance to 12 or 13 antibiotics of the 14 antibiotics employed in this study), comprising one (1) *Salmonella* spp. and one *Campylobacter* spp. Taking into consideration the data on the calculated multiple antibiotic indices of the bacterial isolates, the antibiotic resistance fluctuated over time, showing a reducing trend as presented in Figure 1.

As shown in Table 2, most of the isolates with a MAR index > 0.2 belonged to the genera *Campylobacter* (31.03%) and *Salmonella* (31.03%), followed by *E*. *coli* (20.00%). In addition, a total of ninety-four (94) multidrug resistance phenotypes were observed in the bacterial isolates evaluated against a suite of 14 conventional antibiotics. The distribution of the MDR phenotypes occurred as follows: 29 for *E*. *coli*, 27 for *Campylobacter* spp., 22 for *Salmonella* spp. and 19 for *Yersinia* spp. The most common MDR profiles demonstrated in this study were resistance to five antibiotics (E, TET, SMX, AUG, AMOX) by *Yersinia* spp. and *Salmonella* spp. and resistance to four antibiotics (E, TET, CTX, AMOX) by *E*. *coli* and *Campylobacter* spp. as well as E, AUG, AP and AUG by *Yersinia* spp. and *E*. *coli*. Overall, only one (1) *Campylobacter* spp. demonstrated resistance to the highest number of antibiotics (13) presenting with the MAR phenotype TS, E, C, TET, CIP, SMX, AUG, S, CTX, NA, GM, AP, AMOX. 

Remarkably, all *Yersinia* spp. (100%), *Salmonella* spp. (100%) and 90.63% *E*. *coli* plus 80.36% *Campylobacter* spp. were multidrug-resistant; however, the MAR phenotypes varied with the bacteria as displayed in Table 3. Accordingly, the predominant MAR phenotypes were in the following order: 28.89% (*Salmonella* sp.) presented as TS, CIP, E, C, TET, SMX, AUG, CTX, AMOX, NI; 26.79% (*Campylobacter*) observed as TS, CIP, E, C, TET, SMX, S, CTX, NA, GM; 15.38% (*Yersinia* spp.) associated with the MAR pattern E, SMX, AUG, AP, AMOX; and, lastly, 6.25% (*E*. *coli*) represented by E, TET, CTX, AP, AMOX, NI and E, TET, AUG, CTX, NA, AP, AMOX, NI.

## 4. Discussion

One Health embodies three main components: humans, animals and environment health, emphasising that the health of these three are interdependent or interconnected, meaning any problem facing the health of one component will affect the other two. Accordingly, Mackenzie and Jeggo [35] defined One Health as a collaborative, multisectoral, and transdisciplinary approach, working at the local, regional, national and global levels, with the objective of accomplishing ideal health outcomes, recognising the interconnectedness between people, animals, plants and their shared environment. Antimicrobial resistance (AMR) is viewed as a critical and major One Health problem as it might affect global public health, food safety and food security [14]. The One Health approach is essential in combating antimicrobial resistance as various bacteria, including *E*. *coli*, *Yersinia* spp., *Salmonella* spp. and *Campylobacter* spp., are becoming increasingly resistant and livestock manure may be a reservoir.

Animal manure is viewed as a favourable environment for the survival of pathogens and the number and type of the microbial pathogens occurring in livestock wastes is closely associated with the animal species, physicochemical composition of the manure and the geographic location of the farm, and the feeding habits can determine the biochemical and biological properties of the manures [7]. The level of bacterial counts is a measure of the hygienic condition of the manure; in this study, the bacterial counts ranged from 10^4^ to 10^6^ cfu/mL, with the highest estimated *E*. *coli* counts of 2 × 10^6^ cfu/mL. This can be affirmed by the findings of Dawangpa et al. [36], who mentioned that swine farms have installed water treatment facilities to treat the water prior to discharge into the environment. However, the treated water is being reused on farms after treatment, therefore creating the likelihood that *E*. *coli* could be recycled back to the swine. This could explain the high *E*. *coli* counts enumerated in this study. In addition, the evidence of the varying counts of *E*. *coli*, *Yersinia* spp., *Salmonella* spp. and *Campylobacter* spp. in the manure indicated that the pig manure can be a potential source of contamination to water, soil and agricultural products, thereby causing gastrointestinal infections in children, elderly and immunocompromised individuals [11]. 

Details of the findings on the influence of time on the dynamics of the bacterial species in terms of bacterial counts have been published by Manyi-Loh and Lues [37]. The authors demonstrated further that the different bacterial species were inactivated by 1 log reduction, and *E*. *coli* had the shortest survival period of 77 days, followed by *Salmonella* spp. (84 days) and *Yersinia* spp. (98 days), while *Campylobacter* survived for the longest period of 112 days. This may suggest that the fastidious organisms requiring selective supplements for growth survived for a longer period than *Salmonella* and *E*. *coli* as they had an adequate supply of nutrients encouraging their growth. Moreover, over the period of the study, 56 *Campylobacter* spp., 45 *Salmonella* spp., 32 *E*. *coli* and 26 *Yersinia* spp. were recovered. Although *Yersinia* spp. lasted longer as opposed to *Salmonella* spp. and *E*. *coli*, it can be suggested that the higher number of species of the latter two bacteria could be attributed to the initial concentration of these bacteria in the procured samples [38]. 

The prevalence of the bacteria ranged from 22.22% (4/18; *E*. *coli*) to 50% (9/18; *Campylobacter* spp.), while *Salmonella* sp. and *Yersinia* spp. had a common prevalence rate of 27.78%. Contrary to our findings, Dikonketso and Olayinka [39] enumerated viable cells in seepage samples recovered from a pig farm in the range from 4.30 × 10^2^ to 1.29 × 10^9^ cfu/mL, while Peng et al. [40] noted a prevalence of 2.76% of *Y*. *enterocolitica* in swine faeces collected from Sichuan and Shandong provinces of China. Considering the variation in the bacterial counts and the prevalence rates in manure reported in the different studies, this could be attributed to the fattening stage of the pigs, the sampling times and points [41], the chemical composition (which depends on the feed composition), the type or variety of vegetation available added to the microbiological compositions of the pig manure, environmental temperature as well as the waste collection and management systems on the farms [40].

It is worth mentioning that South Africa is regarded as one of the major contributors to the total global increase in meat consumption [42]. As a consequence, the intensive animal production approach involving frequent and huge applications of antibiotics is adopted to meet the increased demand in meat and meat products. Therefore, the South African Veterinary Association (SAVA) published the guidelines for using antimicrobials in the South African pig industry and advocated for the use of critically and highly important drugs, including streptomycin, gentamicin, erythromycin, ampicillin, ciprofloxacin and tetracycline for pigs, despite their crucial and high relevance in human medicine [43]. With regards to the susceptibility data as shown in Table 1, overall, the bacterial isolates showed diverse susceptibility patterns to all the tested antibiotics, represented by different percentages. The sensitivity displayed, however, depended on the bacterial isolates and the antibiotics used in question: *E*. *coli* (100% amoxicillin; 68.75% chloramphenicol), *Salmonella* spp. (93.33% streptomycin, 68.89% gentamicin), *Yersinia* spp. (96.15% streptomycin, 80.76% ciprofloxacin) and *Campylobacter* spp. (75.00% nitrofurantoin, 71.00% augmentin). These antibiotics are from different classes and as such possess different chemical structures as well as differing modes of action. The findings are similar to those of Musonye and colleagues [44], who mentioned varied susceptibility of the bacterial isolates from urine samples of livestock and wildlife against co-trimoxazole, amoxicillin, ciprofloxacin, streptomycin, nalidixic acid, chloramphenicol and gentamicin. More elaborately, intermediate sensitivity was displayed by 86.67% *Salmonella* sp., 76.92% *Yersinia* sp. and 68.75% *E*. *coli* to nalidixic acid. The demonstration of intermediate sensitivity can be viewed as the bacterial isolates gradually losing sensitivity to this drug; a situation which can be explained by the fact that this drug is normally considered as a strategic therapy against resistant isolates. Therefore, this promoted the wide use of this drug to treat a variety of bacterial diseases in humans, leading to growth in the number of resistant isolates. This is affirmed by the recent study of Moyen et al. [45], who demonstrated a total resistance to nalidixic acid of *Enterobacteriaceae* recovered from household wastewater in Brazzaville, Republic of Congo.

On the other hand, a high and common resistance was shown in all the bacterial isolates to erythromycin, a macrolide. With the exception of the *Campylobacter* isolates, the finding is congruent with the study of Kilonzo-nthenge et al. [46], who reported a total resistance (100%) to erythromycin of bacteria recovered from retailed chicken and beef products that were grouped in the family *Enterobacteriaceae*. In detail, *E*. *coli* isolates in this study demonstrated a considerable resistance to tetracycline (81.25%); consistent with this study were the high resistance rates between 33.3% and 93% reported for tetracycline-resistant *E*. *coli* from dairy farms in many farms operated in the UK, Asia and the USA [47,48]. Clearly, zoonoses are crucial to note, owing to human health. In this light, a significant resistant action was displayed by *Salmonella* spp. to amoxicillin (100%), tetracycline (100%) and sulfamethoxazole (97.78%). This finding is similar to the study of Rasschaert and co-authors [49], who demonstrated that *Salmonella* species from 89 samples of pig manure collected in Flanders (northern part of Belgium) were highly resistant to ampicillin (54.7%), tetracycline (45.3%) and sulfamethoxazole (47.2%). Additionally, 92.13% of *Yersinia* spp. were observed to present with resistance against sulfamethoxazole, contradicting the study of Peng et al. [40], who noted complete resistance (100%) of *Yersinia enterocolitica* to ampicillin, augmentin and cefazolin. Overall, it is inferred that there are differences in the use and management of antibacterial agents on farms between countries [8]. Seemingly, the acquisition and the distribution of antibiotic-resistant genes and bacteria can be affected by a host of factors, including the weather and climate, the breed of the animals, the antibiotic dosage for administration, the duration of the treatment, the capacity of the farm [36], the animal husbandry practices, the hygienic conditions of the farm as well as the commitment with control measures and disease prevention [50]. Additionally, the presence of the carrier animal moving among the animal herds and through vector action is important. 

Nevertheless, our data showing huge resistance to the commonly used antibiotics describes the complex use of antibiotics in pig farming, which employs more antibiotics than poultry and cattle farming and is associated with the interconnecting areas of animal health, welfare and economics [51]. However, there are observed discrepancies in the use of antibiotics across the different stages of pig production, owing to the differences in the diseases, the epidemiology and the route of administration of the available drugs [52]. This could be the reason for the resistance observed to the advocated antibiotics though in varying percentages in the present study; therefore, the findings call for the need for stringent antimicrobial surveillance programmes for the use of antibiotics in animal farming. The members of the *Enterobacteriaceae* exhibited resistance to antibiotics owing to the mobilisation of continuously expressed single genes that encode efficient drug-modifying enzymes. This could be the reason *Salmonella* (100%) isolates in this study demonstrated resistance to amoxicillin. Our findings corroborate those of Singh et al. [53], who reported the resistance to amoxicillin of bacterial isolates recovered from spring water that were categorised as *Enterobacteriaceae*. 

Furthermore, susceptibility to nalidixic acid has been implemented as a criterion to differentiate between *C*. *jejuni* and *C*. *coli*. Only seven percent (7.14%) of the *Campylobacter* isolates were susceptible to nalidixic acid, but 26.79% displayed susceptibility in an intermediate range. On the other hand, *Campylobacter* species demonstrated resistance (66.07%) to nalidixic acid, substantiating the study of Ogbor et al. [54], who noted resistance of a higher magnitude (100%) to nalidixic acid by *C*. *coli* isolated from poultry farms in Lagos Sate, Nigeria. Clearly, the difference in the percentage resistance of *Campylobacter* spp. in both studies can be due to the type of animals investigated (pig against poultry) and the countries in question (South Africa against Nigeria). In detail, Sibanda et al. [55] emphasised that chickens (Avian species) represent a significant reservoir for the transmission of *Campylobacter* species owing to their high body temperature which is necessary for the optimum growth of the pathogen, which can colonise the caeca of chickens in very high numbers. Although a developing country, South Africa is a more advanced country than Nigeria (in terms of GDP) and is governed by more stringent policies relating to antibiotic consumption; however, policies regarding the purchase and consumption of antibiotics in both human and animals will differ between the countries as they have different disease burdens, environmental conditions and socioeconomic status [56]. A host of authors pointed out that the quality of governance, the availability and conditions of health facilities, poverty, education and sanitation are strongly related to the differences observed in antibiotic resistance and antibiotic consumption profiles existing between regions/countries [57,58]. Almost fifty-four percent (53.57 approx. 54%) of the isolates were resistant to ciprofloxacin (another quinolone) and seventy-nine percent (78.57~79%) were resistant to erythromycin, both drugs included in the treatment regimen recommended for the treatment of campylobacteriosis caused by *Campylobacter* species [54]. In addition, the *Campylobacter* isolates established an extreme resistance (98.12%) to cefotaxime. Taking into consideration the above-mentioned prevalence of resistance, public health attention is aroused because the organism is considered as one of the key zoonotic pathogens responsible for the cause of gastroenteritis in humans worldwide [59].

The multiple antibiotic resistance (MAR) index describes resistance to three or more antibiotics. Monitoring of resistance to multiple antibiotics is critically necessary for effective containment programmes, and more especially in Gram-negative bacteria, which is the case with this study. Taking into consideration the data on the calculated multiple antibiotic indices of the bacterial isolates, the antibiotic resistance fluctuated over time as presented in Figure 1. The effect of time on the bacterial antibiotic resistance seems to mirror the growth curve of a bacterium. In any environment that a bacterium is found, it tends to thrive by first adapting to the environmental conditions, then grows and multiplies via binary fission (the parent bacterium produces a replica of itself, including antibiotic resistance genes), thus causing a rise in antibiotic resistance genes. An increase in the antibiotic resistance could also be attributed to bacterial isolates taking up resistance genes through horizontal gene transfer mediated by integrons, transposons, plasmids via transduction, transformation and conjugation [60]. However, when growth of the bacterial cells become limited, cell division ceases and the bacterial cells tend to die out, encountering a decline phase [61] owing to unfavourable conditions, including limited space, exhaustion of nutrients and the accumulation of toxic wastes/substances in the environment. These conditions have been reported to occur in an anaerobic digester as the process progresses with time. Accordingly, Jiang et al. [62] noted a combination of factors causing bacterial reduction via anaerobic digestion in a biodigester. Therefore, from Figure 1 it can be depicted that the antibiotic resistance was reduced over time. The findings corroborate those of Katada et al. [63] who noted that antibiotic resistance genes such as *tet A*, *tetB* and *bla _TEM_* were reduced through the anaerobic treatment of livestock manure.

Clearly, a biodigester, a bioengineered environment, equally creates an impact on the trend of antibiotic resistance of the bacteria present; however, the extent of this effect will depend on whether the digester is continuous or batch-operated. This is because fresh substrates are added intermittently and digestate is discharged simultaneously in the continuous mode, affecting both the microbial population and antibiotic resistance as the microbial composition of the added substrate can influence the antibiotic resistance genes content of the digester [60] as the inactivation of the bacterial population will not be efficient since the starting bacterial population is not allowed to spend substantial time inside the digester. Contrarily, in batch operation, which is the case with this study, the substrates were fed once into the digester and were discharged only after the anaerobic digestion process was completed, thus leading to the reduction of the bacterial isolates as presented by Manyi-loh and Lues [37] as well as the antibiotic resistance.

The study revealed a very high prevalence of 91.19% (145/159) of MAR bacterial isolates exhibiting resistance to ≥3 antibiotics and MAR indices > 0.2, indicating that the MAR bacteria arose from a probably hazardous source where antibiotics are used often, such as in the pig farm, either for growth promotion, treatment or prevention of diseases [8] as well as the occurrence of high selective pressure in this environment. Varying levels of resistance and MDR were noted in relation to the different antibiotic classes tested against the bacterial isolates. The display of different MDR phenotypes in different permutations and combinations provides the evidence of the complexity and diversity of resistance in animal manure [27], which has been considered as a reservoir of pathogenic bacteria, resistant bacteria and resistance genes. The demonstration of resistance to the antibiotics recommended by SAVA indicated the possibility of high exposure of these organisms on the farm. 

A spectacular fraction of the bacterial isolates (91.19%) were MDR with *Yersinia* spp., *Salmonella* spp., *E*. *coli* and *Campylobacteria* spp. recording 100%, 100%, 90.63% and 80.36%, respectively (Table 3). As shown in Table 3, overall, the MDR bacterial isolates displayed a total of 94 MAR phenotypes, with *E*. *coli* demonstrating more diverse resistance profiles (29 MDR phenotypes), followed by *Campylobacter* spp. (27 MDR phenotypes). In accordance with our findings was the 95.5% resistance to three or more classes of antimicrobials noted by Chala et al. [64] of bacterial isolates recovered from humans, animals and water sources in livestock-owning households, residing in the peri-urban areas of Addis Ababa, Ethiopia. It is striking and a call for concern that almost all the isolates belonging to *Enterobacteriaceae* (*Yersinia* spp., *Salmonella* spp. and *E*. *coli*) were multidrug-resistant.

In addition, two (2) isolates presented with a MAR index of 0.9, showing resistance to almost all the antibiotics employed in the investigation and also higher selective pressure. Therefore, these strains offer higher chances of contamination and the spread of antibiotic resistance genes (serving as vehicles for antibiotic resistance genes) via horizontal gene transfer. Moreover, one (1) *Campylobacter* isolate showed a MAR phenotype of TS, E, C, TET, CIP, SMX, AUG, S, CTX, NA, GM, AP, AMOX, insinuating resistance to 13 tested antibiotics. Thus, Ogbor et al. [54] opined that there seems to be a paradigm shift, with *Campylobacter* isolates now becoming multidrug-resistant, notably, in their resistance to tetracycline and fluoroquinolone. According to the One Health concept, our findings of antibiotic-resistant and multidrug-resistant bacterial pathogens would have serious implications in humans when the untreated manure is applied on agricultural lands and ultimately enters ground water and spring water utilised by humans for domestic and sanitation purposes via hydrological processes (heavy rainfall or storms), creating opportunities for diseases and infections [6]. The diseases and infections caused by bacteria expressing decreased sensitivity to the highly recommended drugs employed for antimicrobial chemotherapy, therefore, result in failed treatment, the patient’s condition becoming deteriorated as well as causing elevated financial constraints on the people and the facility engaged in the delivery of health care services [15]. Additionally, humans might ingest these resistant bacteria from meat that is contaminated with faeces/manure during slaughtering at the abattoirs. The higher prevalence of MDR *Enterobacteriace* (Gram-negative bacilli) can cause difficult to treat infections, consequently endangering a greater number of hospitalised patients [15], interfering with many facets of antimicrobial stewardship. Similarly, the observation of high levels of resistance and multidrug resistance is rather disturbing, considering that South Africa has a high prevalence of HIV/AIDS, a population who depend on the use of antibiotics to boost their immune system during the management of more than a few bacterial infections, including gastrointestinal problems that are recurrent in the said population [22]. In addition, spillover of the resistant pathogens and resistance genes can occur from the faeces of one animal to another via horizontal transfer as these animals live in proximity on the farm [27]. 

However, in this study, remarkable resistance was shown against erthromycin, amoxicllin, sulfamethoxazole, tetracycline and cefotaxime. Future studies are inevitable to ascertain the occurrence of the resistance genes provoking the resistance action in the bacterial isolates recovered from this study, and virulence factors will be performed simultaneously since they are interconnected. 

## 5. Conclusions

Clearly, the pig manure has been shown to contain zoonotic enteropathogens in varying levels or counts with maximum counts due to *E*. *coli*. This study ascertained the presence of multidrug-resistant bacteria in a mixture of pig manure and pine wood saw dust. The results showed that the bacterial isolates demonstrated great resistance to erythromycin, sulfamethoxazole, amoxicillin, tetracycline and cefotaxime. Additionally, the MAR index occurred between 0.1 and 0.9, with 91.19% of the isolates exhibiting resistance to ≥3, a situation that merits public health attention. This is so because some of the tested bacteria are the major waterborne or foodborne pathogens, which are capable of causing close to 2 million deaths per year in developing countries. Having the resistant counterpart of these bacteria will apparently compound the problem further, posing a serious threat to health care systems as the organisms spread from the environment to clinical settings. 

Acknowledging the fact that antimicrobial resistance is a growing global problem, it is therefore pertinent to conduct periodic monitoring of resistance patterns of common bacteria of public health and environmental significance to receive updates on their susceptibility/resistance profiles that might in turn be beneficial to the patient as well as clinician in the selection of chemotherapy. The profile of antibiotic resistance fluctuates with socioeconomic strata, geographic criteria and it is different between studies; bacterial resistance is affected by the time span, the study design and the type of population involved in the investigation [17]. Nonetheless, following the findings of this study presenting the high prevalence of resistance to erythromycin, sulfamethoxazole, amoxicillin and tetracycline, it is worth concluding that the individual antibiotics should not be used as a monotherapy as our results clearly reject the antibiotics to be employed as the empirical treatment of infections caused by these bacterial isolates and for effective hospital infection control. Additionally, the pig manure tested should not be employed as a fertiliser according to traditional custom unless treated further, as it will serve as a source of contamination and dissemination of genes resistant to antibiotics to the microbial population in the environment and clinical setting. In addition, the findings represent a baseline for future investigations into identifying the genes responsible for antibiotic resistance and or virulence in these organisms in order to devise alternative therapies in the form of vaccines and antimicrobials to prevent and cure infections.

## Figures and Tables

**Table 1:** Frequency of antibiotics’ susceptibility patterns among the bacterial isolates in this study.

Class of Antibiotics	Examples of Antibiotics	Total Number of Bacterial Isolates Investigated in This Study, *n* = 159											
		*Yersinia* spp. (*n* = 26)			*Escherichia coli* (*n* = 32)			*Salmonella* spp. (*n* = 45)			*Campylobacter* spp. (*n* = 56)		
		Sensitive	Intermediate	Resistant	Sensitive	Intermediate	Resistant	Sensitive	Intermediate	Resistant	Sensitive	Intermediate	Resistant
Sulphonamides	Trimethoprim- Sulfamethoxazole (Co-trimoxazole)	16 (61.54)	3 (11.54)	7 (26.92)	20 (62.50)	3 (9.38)	9 (28.13)	7 (15.56)	2 (4.44)	36 (80.00)	31 (55.36)	2 (3.57)	23 (41.07)
	Sulfamethoxazole	0 (0.00)	2 (7.69)	24 (92.31)	0 (0.00)	14 (43.75)	18 (56.25)	1 (2.22)	0 (0.00)	44 (97.78)	25 (44.64)	1 (1.79)	30 (53.57)
Quinolones	Ciprofloxacin	21 (80.76)	3 (11.54)	2 (7.69)	13 (40.63)	14 (43.75)	5 (15.63)	1 (2.22)	16 (35.56)	28 (62.22)	17 (30.36)	9 (16.07)	30 (53.57)
	Nalidixic acid	4 (15.38)	20 (76.92)	2 (7.69)	4 (12.50)	22 (68.75)	6 (18.75)	2 (4.44)	39 (86.67)	4 (8.89)	4 (7.14)	15 (26.79)	37 (66.07)
Macrolides	Erythromycin	0 (0.00)	0 (0.00)	26 (100)	0 (0.00)	3 (9.38)	29 (90.63)	0 (0.00)	0 (0.00)	45 (100)	2 (3.57)	10 (17.86)	44 (78.57)
Phenicols	Chloramphenicol	8 (30.77)	8 (30.77)	10 (38.46)	22 (68.75)	5 (15.63)	5 (15.63)	7 (15.56)	0 (0.00)	38 (84.44)	17 (30.36)	2 (3.57)	37 (66.07)
Tetracyclines	Tetracycline	9 (34.62)	5 (19.23)	12 (46.15)	4 (12.50)	2 (6.25)	26 (81.25)	0 (0.00)	0 (0.00)	45 (100)	19 (33.93)	7 (12.5)	30 (53.57)
Penicllins	Amoxicillin-Clavulanate (Augmentin)	4 (15.38)	0 (0.00)	22 (84.62)	1 (3.13)	14 (43.77)	17 (50.13)	0 (0.00)	9 (20.00)	36 (80.00)	40 (71.43)	12 (21.43)	4 (7.14)
	Ampicillin	4 (15.38)	4 (15.38)	18 (69.23)	2 (6.25)	7 (21.88)	23 (71.88)	8 (17.78)	29 (64.44)	8 (17.78)	38 (67.86)	11 (19.64)	7 (12.5)
	Amoxicillin	1 (3.85)	2 (7.69)	23 (88.46)	32 (100)	0 (0.00)	0 (0.00)	0 (0.00)	0 (0.00)	45 (100)	23 (41.07)	9 (16.07)	24 (42.86)
Aminoglycosides	Gentamicin	20 (76.92)	5 (19.23)	1 (3.85)	12 (37.50)	14 (43.75)	6 (18.75)	31 (68.89)	12 (26.67)	2 (4.44)	22 (39.29)	2 (3.57)	32 (57.14)
	Streptomycin	25 (96.15)	1 (3.85)	0 (0.00)	21 (65.63)	10 (31.25)	1 (3.13)	42 (93.33)	3 (6.67)	0 (0.00)	26 (46.43)	3 (5.36)	27 (48.21)
Nitrofurans	Nitrofurantoin	12 (46.15)	12 (46.15)	2 (7.69)	5 (15.63)	10 (31.25)	17 (53.13)	2 (4.44)	11 (24.44)	32 (71.11)	42 (75.00)	2 (3.57)	12 (21.43)
Cephalosporins	Cefotaxime	8 (30.77)	12 (46.15)	6 (23.08)	0 (0.00)	9 (28.13)	23 (71.88)	1 (2.22)	3 (6.67)	41 (91.11)	0 (0.00)	1 (1.79)	55 (98.21)

*n*, total number of a particular bacterial organism.

**Table 2:** Distribution of the bacterial isolates depending upon MAR > 0.2.

Bacterial Isolates	Range of MAR Indices	Number of Bacterial Isolates with MAR > 0.2 (*n* = 145)	Percentage (%)
*Yersinia* spp.	0.3–0.7	26	17.93
*E*. *coli*	0.1–0.8	29	20.00
*Salmonella* spp.	0.4–0.9	45	31.03
*Campylobacter* spp.	0.1–0.9	45	31.03
Total		145	100

**Table 3:** Resistance patterns observed in the multidrug-resistant bacterial isolates.

	*Yersinia* spp. (*n* = 26)	
No. of Antibiotics	MAR Phenotypes (*n*= 19)	Frequency of Isolates in Percentages (%)
5	E, AUG, AP, AMOX, SMX	4 (15.38%)
5	E, AUG, AP, AMOX, NA	1 (3.85%)
6	E, AUG, AP, AMOX, SMX, NI	2 (7.69%)
6	E, SMX, TET, TS, C, NI	2 (7.69%)
6	E, AUG, AP, AMOX, SMX, NI	1 (3.85%)
6	E, AUG, AP, AMOX, SMX, NA	1 (3.85%)
6	E, AP, AMOX, SMX, CTX, CIP	1 (3.85%)
7	E, AUG, AP, AMOX, SMX, CTX, C	1 (3.85%)
7	E, AUG, AP, AMOX, SMX, NI, CIP	1 (3.85%)
7	E, SMX, TET, TS, C, NI, GM	1 (3.85%)
7	E, AUG, AP, AMOX, SMX, CTX, NI	1 (3.85%)
7	E, AUG, AP, AMOX, SMX, TET, NI	1 (3.85%)
7	E, AUG, AMOX, SMX, TET, TS, C	1 (3.85%)
8	E, AUG, AP, AMOX, SMX, TET, NI, C,	2 (7.69%)
8	E, AUG, AP, AMOX, SMX, TET, TS, C	1 (3.85%)
8	E, AUG, AMOX, SMX, CTX, TET, TS, C	1 (3.85%)
10	E, AUG, AP, AMOX, SMX, CTX, TET, TS, C, NI	1 (3.85%)
Total of MDR isolates		26
Percentage MDR		100%
	***Escherichia coli*** **(spp.) (*n* = 32)**	
**No. of Antibiotics**	**MAR Phenotypes (*n* = 29)**	**Frequency of Isolates in Percentages (%)**
3	AMOX, TET, AP	1 (3.13%)
3	AMOX, TET, CTX	1 (3.13%)
4	E, AMOX, SMX, TET	1 (3.13%)
5	E, AMOX, CTX, TET, NI	1 (3.13%)
5	E, AUG, AP, AMOX, TET	1 (3.13%)
6	E, AP, AMOX, SMX, CTX, NA	1 (3.13%)
6	E, AUG, AP, AMOX, CTX, TET	1 (3.13%)
6	E, AMOX, SMX, CIP, NA, GM	1 (3.13%)
6	E, AP, AMOX, CTX, TET, NI	2 (6.25%)
6	E, AMOX, SMX, CTX, TS, C	1 (3.13%)
6	E, AP, AMOX, SMX, TET, NI	1 (3.13%)
7	E, AUG, AP, AMOX, SMX, CTX, TET	1 (3.13%)
7	E, AUG, AP, AMOX, CTX, TET, NI	1 (3.13%)
7	E, AP, AMOX, SMX, CIP, GM, NI	1 (3.13%)
8	E, AUG, AP, AMOX, SMX, CTX, TET, TS	1 (3.13%)
8	E, AUG, AP, AMOX, CTX, TET, NA, NI	2 (6.25%)
8	E, AUG, AP, AMOX, SMX, CTX, TET, CIP	1 (3.13%)
8	E, AUG, AP, AMOX, SMX, CTX, TET, NI	1 (3.13%)
8	E, AUG, AP, AMOX, CTX, TET, CIP, NI	1 (3.13%)
8	E, AUG, AP, AMOX, SMX, TET, NI, TS	1 (3.13%)
9	E, AMOX, SMX, CTX, TET, NI, TS, C, GM	1 (3.13%)
9	E, AUG, AP, AMOX, SMX, CTX, NI, C, TS	1 (3.13%)
9	E, AUG, AP, AMOX, SMX, CTX, TET, NA, NI	1 (3.13%)
10	E, AUG, AP, AMOX, SMX, CTX, TET, TS, S, GM	1 (3.13%)
10	E, AMOX, SMX, CTX, TET, NA, NI, GM, C, TS	1 (3.13%)
10	E, AUG, AP, AMOX, SMX, CTX, TET, TS, NI, CIP	1 (3.13%)
11	E, AUG, AP, AMOX, SMX, CTX, TET, TS, NI, C, GM	1 (3.13%)
Total of MDR isolates		29
Percentage MDR		90.63%
	***Salmonella* spp. (*n* = 45)**	
**No. of Antibiotics**	**MAR Phenotypes (*n* = 22)**	**Frequency of Isolates in Percentages (%)**
5	E, AMOX, TET, NA, NI	1 (2.22%)
6	E, AUG, AMOX, SMX, TET, NI	2 (4.44%)
7	E, AUG, AMOX, SMX, CTX, TET, NI	2 (4.44%)
7	E, AMOX, SMX, CTX, TET, TS, C	1 (2.22%)
8	E, AMOX, SMX, CTX, TET, TS, C, CIP	3 (6.67%)
8	E, AUG, AMOX, SMX, CTX, TET, TS, C	1 (2.22%)
9	E, AUG, AMOX, SMX, CTX, TET, TS, C, NI	4 (8.89%)
9	E, AUG, AMOX, SMX, CTX, TET, TS, C, CIP	2 (4.44%)
9	E, AMOX, SMX, CTX, TET, TS, C, CIP, NI	2 (4.44%)
9	E, AP, AMOX, SMX, CTX, TET, TS, C, CIP	1 (2.22%)
9	E, AUG, AP, AMOX, SMX, CTX, TET, TS, C	1 (2.22%)
9	E, AUG, AMOX, SMX, CTX, TET, TS, C, NI	2 (4.44%)
9	E, AUG, AP, AMOX, SMX, CTX, TET, C, NI	1 (2.22%)
9	E, AMOX, SMX, CTX, TET, TS, C, CIP, NA	1 (2.22%)
9	E, AUG, AMOX, SMX, CTX, TET, CIP, GM, NI	1 (2.22%)
10	E, AUG, AMOX, SMX, CTX, TET, TS, C, CIP, NI	13 (28.89%)
10	E, AUG, AP, AMOX, SMX, CTX, TET, TS, C, CIP	2 (4.44%)
10	E, AUG, AP, AMOX, SMX, CTX, TET, CIP, C, NI	1 (2.22%)
11	E, AUG, AMOX, SMX, CTX, TET, TS, C, CIP, NA, NI	1 (2.22%)
11	E, AUG, AP, AMOX, SMX, CTX, TET, TS, C, GM, NI	1 (2.22%)
12	E, AUG, AP, AMOX, SMX, CTX, TET, TS, C, CIP, NA, NI	1 (2.22%)
Total MDR isolates		45
Percentage MDR		100%
	***Campylobacter* spp. (*n* = 56)**	
**No. of Antibiotics**	**MAR Phenotypes (*n* = 27)**	**Frequency of Isolates in Percentages (%)**
3	E, CTX, AMOX	3 (5.36%)
3	E, CTX, NA,	1 (1.79%)
3	AMOX, CTX, AUG	1 (1.79%)
3	AMOX, CTX, NA	2 (3.57%)
4	AMOX, CTX, TET, AP	1 (1.79%)
5	CTX, C, NI, E, AMOX	4 (7.14%)
5	CTX, C, NI, E, SMX,	1 (1.79%)
5	CTX, C, NI, AP, AMOX	2 (3.57%)
5	CTX, CIP, NA, GM, SMX	1 (1.79%)
6	CTX, CIP, NA, GM, E, C	2 (3.57%)
6	CTX, C, NI, AP, AMOX, E	2 (3.57%)
6	E, CTX, CIP, NA, GM, S	1 (1.79%)
6	E, CTX, CIP, NA, GM, SMX	1 (1.79%)
6	CTX, C, NI, E, AMOX, NA	1 (1.79%)
6	CTX, S, NI, AMOX, AUG, TET	1 (1.79%)
8	E, SMX, CTX, TET, C, NA, GM, TS	1 (1.79%)
8	E, SMX, CTX, TET, C, NA, GM, S	1 (1.79%)
8	E, SMX, CTX, TET, AMOX, NA, GM, S	1 (1.79%)
8	E, SMX, CTX, TET, CIP, NA, GM, TS	1 (1.79%)
9	E, SMX, TET, CIP, NA, GM, TS, S, C	1 (1.79%)
9	E, SMX, CTX, TET, CIP, NA, GM, S, C	1 (1.79%)
10	E, SMX, CTX, TET, CIP, NA, GM, S, C, TS	15 (26.79%)
11	E, SMX, CTX, TET, CIP, NA, GM, S, C, TS, AMOX	3 (5.36%)
11	E, SMX, CTX, TET, CIP, NA, GM, S, C, TS, NI	1 (1.79%)
11	E, SMX, CTX, TET, CIP, NA, GM, S, C, TS, AUG	1 (1.79%)
13	E, SMX, CTX, TET, CIP, NA, GM, S, C, TS, AUG, AP, AMOX	1 (1.79%)
Total MDR isolates		45
Percentage MDR		80.36%

TS, Co-trimoxazole; E, Erythromycin; TET, Tetracycline; CIP, Ciprofloxacin; SMX, Sulfamethoxazole; AUG, Augmentin; S, Streptomycin; CTX, Cefotaxime; NA, Nalidixic acid; GM, Gentamicin; AP, Ampicillin; AMOX, Amoxicillin; NI, Nitrofurantoin.

## Data Availability

Data available on request due to restrictions (privacy). The data presented in this study are available on request from the corresponding author. The data are not publicly available as the Intellectual property belongs to the University.
